# Global Impact of COVID-19 Pandemic on Physical Activity Habits of Competitive Runners: An Analysis of Wearable Device Data

**DOI:** 10.3390/ijerph191912933

**Published:** 2022-10-10

**Authors:** Julia Lee Romero, Qin Lv

**Affiliations:** Department of Computer Science, University of Colorado Boulder, Boulder, CO 80309, USA

**Keywords:** wearable device, physical activity, pandemic policy, COVID-19, pandemic, exercise habits, running, objectively measured physical activity

## Abstract

The COVID-19 pandemic resulted in government restrictions that altered the lifestyle of people worldwide. Studying the impact of these restrictions on exercise behaviors will improve our understanding of the environmental factors that influence individuals’ physical activity (PA). We conducted a retrospective analysis using an stringency index of government pandemic policies developed by Oxford University and digitally-logged PA data from more than 7000 runners collected using a wearable exercise-tracking device to compare the relationship between strictness of lockdowns and exercise habits on a global scale. Additionally, the time-of-day of PA globally, and activity-levels of PA in 14 countries, are compared between the pre-pandemic year of 2019 and the first pandemic year of 2020. We found that during the pandemic year there was a major shift in the time-of-day that runners exercised, with significantly more activity counts logged during standard working hours on workdays (*p* < 0.001) and fewer activities during the same time frame on weekends (*p* < 0.001). Of the countries examined, Italy and Spain had among the most strict lockdowns and suffered the largest decreases in activity counts, whereas France experienced a minimal decrease in activity counts despite enacting a strict lockdown with certain allowances. This study suggests that there are several factors affecting PA of dedicated runners, including government policy, workplace policy, and cultural norms.

## 1. Introduction

The COVID-19 pandemic impacted the lives and well-being of people worldwide. Early in 2020, the COVID-19 pandemic disrupted the normal lifestyle of people around the world, due to government-issued lockdowns which required people to spend significant time sheltering inside their homes. Countries instituted stay-at-home orders that resulted in transitioning from traditional in-person schooling and work to remote modalities, including travel bans, non-essential workplace closures, and restricting public gatherings. Restrictions caused social isolation, affecting mental and physical health [1]. There is evidence that the psychological impact of the pandemic was strongly negative and lasted throughout the entirety of the 2020 pandemic year and continued to decline even after the pandemic was brought under better control [2]. Additionally, there is concern that the sustained loss of access to parks and fitness centers might have resulted in an increase in sedentary behavior [3]. After the pandemic was brought under better control, towards the end of 2020, the lifting of restrictions resulted in a return to normality in many aspects. Reopening public parks and fitness centers has allowed for a return to prior physical activity (PA) behavior, however, many factors such as masking requirements, fear of contracting the virus, and capacity limitations likely discouraged some people from returning to normal behavior. Furthermore, there are open questions regarding how long-term human behavior was affected from the pandemic.

Physical activity provides a range of benefits for overall well-being including alleviating stress and depression, maintaining a healthy body composition, improving sleep quality, and mitigating certain types of disease. Investigating the impact of the pandemic on physical activity behavior could provide valuable information for researchers, public health officials, policymakers, and employers to better understand and promote healthy PA during the remainder of this pandemic, future pandemics, and during normal, pandemic-free times. Furthermore, this pandemic provided an unprecedented opportunity to use sensing technology not available in previous pandemics to collect data on how people responded to conditions caused by the pandemic. This large-scale digital data on exercise habits holds potential to elucidate the policy, environmental, and personal influences that drive healthy lifestyle choices [3,4].

A majority of previous research on PA during the COVID-19 pandemic found that PA generally decreased [5,6]. These studies used a variety of data sources including survey questionnaires, digital step-counts, and internet search trends, and have yielded inconclusive insights into PA levels during the pandemic due to the heterogeneity of data sources, types of PA tracked (i.e., step-counts, active minutes, intensity of exercise), and subject population [5,6]. Stockwell et al. published a systematic review in early 2021 that included 45 papers reporting the changes in PA from before to during the COVID-19 pandemic; only four of these papers reported device-based measures and the rest based on questionnaires [5]. Most research on PA uses self-reported survey data, which is subject to recall bias compared with objectively-measured activity data [5,7,8]; furthermore, subjects are more likely to over or underestimate their PA when self-reporting [9,10]. Detailed interpretation of PA from questionnaires are discouraged [9]. Objectively-tracked PA data is more sensitive than surveying [9] and thus the literature highlights the need for more behavioral research based on objective measures in order to provide direct accurate comparisons [5,6,9,10]. There remains a large gap between questionnaire-based studies and objective studies using activity tracking devices.

Objective data can also be misleading because it can fail to capture true activity. Studies using smartphone activity-tracking data found that both step-counts and active time decreased during the 2–3 months following initial lockdowns [11,12]. While this research indicates that step-counts may have decreased, big data analyses suggest an increase in other measures of PA [13,14,15,16]. Fitbit, a large wearable device company, reported that overall steps were down from 2019 to 2020, however, active minutes increased for 42% of Fitbit users from 2019 to 2020 [15]. Strava, a leading exercise-recording platform that reached 73 million users in 2020, reported that from 2019 to 2020 global activity counts increased 33% and global outdoor running activities increased 90% [16]. In summary, while multiple sources of data suggest that step-counts declined, big data analysis from wearable device platforms suggests that people were logging dedicated exercise efforts more frequently than prior to the pandemic, perhaps in an effort to compensate for reduced daily-steps.

There are several studies examining a particular country’s exercise habits during initial lockdown periods [11,17,18]; or evaluating exercise habits of particular groups like disabled adults [19,20], older adults [21], working parents [22]; or using survey data to compare between active and infrequent exercisers, [23,24]. Ding et al. reports survey results spanning 11 countries and 11,775 subjects, showing that residents of countries with more stringent COVID-19 response policies were more likely to be insufficiently active according to public health recommendations [18]. While these studies are useful for understanding PA in particular groups, there is limited research directed at investigating governmental pandemic policies in coordination with physical activity in multiple countries. Research leveraging large objective datasets will improve understanding of PA during the COVID-19 pandemic.

In this work, we conduct a exploratory data analysis of wearable device data recorded internationally over a two-year period including a pre-pandemic year (2019) and a pandemic year (2020), using data obtained from the running wearable device company Stryd (www.stryd.com; accessed on 6 October 2022). Our work includes descriptive and statistical analyses and elucidates the PA behaviors of dedicated runners, including time of exercise and activity levels, and compares them between years, countries, and severity of government COVID-19 containment policies. Specific contributions of this work include the following:

- This work employs objective activity-tracking data from a wearable device to provide a direct comparison of running exercise behavior of dedicated runners between a pre-pandemic year and a COVID-19 pandemic year.
- We elucidate how PA changes in dedicated runners at the country level and provide descriptive comparison between countries and pre-pandemic to pandemic years. We discuss specific pandemic policies enacted by countries and the observed PA of runners.
- We show that from the pre-pandemic to the pandemic year there were several large and significant shifts in the time of day that runners log activity, and we break these patterns down by country.

## 2. Materials and Methods

### 2.1. Description of Wearable Device

Running activity data was provided by the company Stryd, which markets a footpod for runners and maintains an online analytics dashboard and smartphone application for users to track their training. The Stryd footpod attaches to shoelaces and can be connected via Bluetooth to a smartphone or fitness watch.

Stryd’s footpod is not used for continuous daily tracking; it is used only for in-run tracking. Stryd’s footpod costs $219 in early 2020 and is marketed towards dedicated runners with an interest in technically-detailed fitness data. Due to its price-point and marketing, Stryd users tend to be dedicated amateur runners, competitive runners, college, semi-professional, and professional runners, or triathletes. As such running is likely to be among the highest priority types of exercise for these users.

### 2.2. Data Cleaning

**Figure 1:**
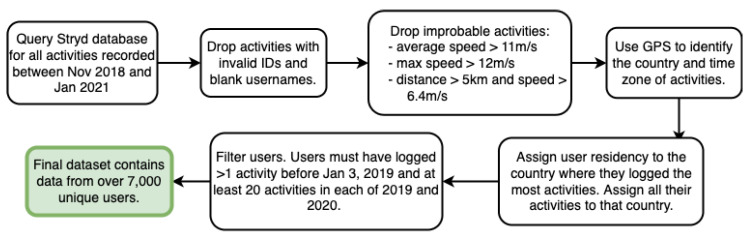
Overview of data cleaning steps.

We obtained anonymized data collected from 1 November 2018 through 1 January 2021 according to Coordinated Universal Time (UTC). All activities represent runs and include fields such as unique user IDs, timestamps, distance traveled, elapsed time during the activity, average speed, local temperature, and starting Global Positioning System (GPS) coordinates. Some activities lack GPS coordinates due to setting configurations or if performed on a treadmill. We clean the data as shown in Figure 1. Initial cleaning was performed, including dropping rows with blank usernames or without valid identifiers. A sample of the first author’s personal data is displayed in Table S1 in Supplementary Materials.

For our analysis, only users who had logged a run on or prior to 3 January 2019 (UTC) were kept, all others were dropped. Near world-record caliber activities were dropped (0.78% of activities), defined as having average speeds exceeding 11 m/s, max speed exceeding 12 m/s, or having a combination of individual activity distance greater than 5000 m with an average speed greater than 6.4 m/s.

GPS coordinates were used to map activities to country and time zone with the Python packages timezonefinder and reversegeocode. Timestamps were converted from UTC time zone to the local time zone. 18.3% of 2019 activities and 0.7% of 2020 activities were without GPS data, due to advances in the Stryd platform’s compatibility and also perhaps due to decreased indoor exercise during COVID-19. To handle this difference in the number of activities lacking GPS data between 2019 and 2020, non-GPS runs were assigned to the country and time-zone where the majority of the individual’s runs with GPS data took place. Users without a single majority country over the two years and users with different majority countries between 2019 and 2020 were dropped from the analysis.

Users who had logged at least one activity before 3 January 2019 and at least 20 activities in each of 2019 and 2020 were included. After the cleaning steps, over 7000 unique users remained in our dataset. For the country specific analysis we select the 14 countries with the most Stryd users. The number of users within the 14 countries that we analyzed ranges from over 90 users to over 2000 users.

### 2.3. Analysis of Exercise Behaviors

#### 2.3.1. Activity Counts

In this paper, activity counts are defined as the number of activities recorded in Stryd’s database. We examine activity counts for different time periods and countries.

#### 2.3.2. Government Policy Strictness

Government policy strictness was quantified using Oxford’s Government Response Stringency Index (GRSI), a time-series government policy strictness index for individual countries capturing the harshness of several virus containment strategies including school, workplace, and public transport closures, cancellations of public events and gathering restrictions, stay-at-home requirements, restrictions on internal and international travel, and public information campaigns [25]. The GRSI is on a scale of 0 to 100, with 0 indicating a lack of pandemic policy, and can be directly compared across countries. Our analysis employs the GRSI in order to explore physical activity changes in the pandemic year that correspond with major policy changes.

#### 2.3.3. Activity Counts by Country

We selected 14 countries that are highly represented in our dataset. First, we examine total activity counts by year and country. The World Health Organization (WHO) declared COVID-19 as a global pandemic on 11 March 2020. To control for seasonality, we compare and report the change in activity counts from 2019 to 2020 using the 67-day periods before and after 11 March. Since 2020 is a leap year, we remove 29 February data from the analysis to keep the length of the time periods equal. We also plot the distribution of the number of activities logged in a year by each user, broken down by country and pre-pandemic and pandemic year.

We also conduct a time-series analysis of activity counts. We reported normalized activity counts by day and country. Normalized activity counts were calculated by dividing the number of activities that day in the given country by the number of users in the country. This normalization allows activity counts to be compared between countries, since the number of users differed in each country. Finally, a 14-day rolling average was applied to the activity counts to smooth out variations due to weather and weekday.

#### 2.3.4. Time of Day That Athletes Run

Time of day of activities was calculated using GPS coordinates to identify the respective time zones and extract local time. To examine pandemic-related behavior, we restricted this analysis to data recorded after March 11th in both years, since 11 March 2020 was the WHO pandemic declaration date.

Activities were partitioned into one-hour bins for each hour of a 7-day week. Activity counts were normalized by dividing each bin activity count by the largest bin activity count. Then, the difference in activity counts in 2019 and 2020 were plotted for each bin.

Next, we defined three time periods: before work (12 AM to 8 AM), during standard working hours (8 AM to 5 PM), and after work (5 PM to 12 AM). These time periods are limited in that they do not represent all job types. Activity counts for each time period on each day were calculated then normalized equally by dividing by the largest bin size.

We hypothesized that the mass shift to working- and schooling-from-home provided more schedule flexibility. We conducted 2-tailed student’s t-tests paired by user to test for the difference between mean activity counts per time bin per day between 2019 and 2020. A test is conducted individually for each of the time bins on each day, comparing the binned activity counts of each user between years. This test is also conducted for each of the 14 selected countries, individually. We use Python’s SciPy package for this statistical analysis.

## 3. Results

### 3.1. Exercise Habits by Country and Government Response

#### 3.1.1. Activity Frequency by Country

**Figure 2:**
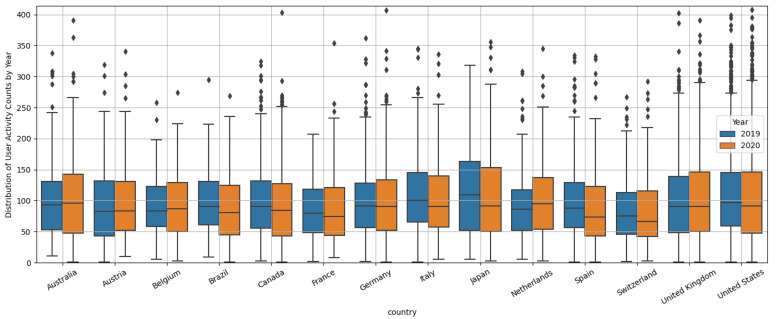
Distribution of user activity counts broken down by country and year.

**Figure 3:**
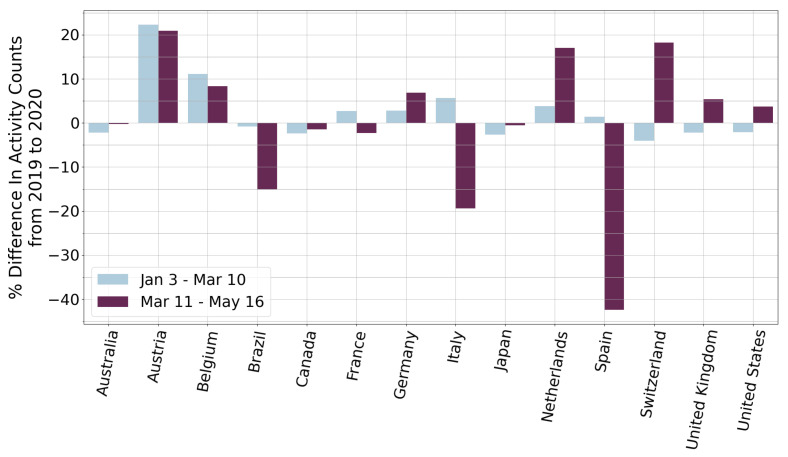
Percent difference in activity counts from 2019 to 2020 compared for the 67-day periods before and after the March 11 World Health Organization (WHO) pandemic declaration date.

There were 4.11% fewer running activities logged in 2020 than in 2019 for all countries in aggregate. Figure 2 shows a comparison between country and year of user activity counts. There is variation across countries in how the median and interquartile range of activity count per user changes from the pre-pandemic to pandemic year, with 8 countries having a decreased median, 3 countries having an increased median, and the others having a very similar median. Similarly, most countries had an increased interquartile range, suggesting that PA behavior of users diverged during the pandemic. Figure 3 shows the difference in activity counts from 2019 to 2020 for 14 countries, corresponding to the two 67-day periods before and after 11 March, the date the WHO declared a pandemic. This data is tabulated in Supplementary Materials (Table S1). Additionally, Table S3 in Supplementary Materials shows the time range and overview of the most restrictive policies enacted in these countries. The countries with the largest decrease in activity between before and after 11 March are Spain, Italy, and Brazil, all with decreases larger than 14%. This decrease indicates that PA of residents of these countries may have been more greatly affected by the pandemic and that these countries’ lockdown policies may be detrimental to maintaining PA. Italy and Spain had very restrictive stay at home orders with Italy and Spain not allowing any outdoor exercise (Table S3). Brazil did not impose any restrictions on outdoor exercise that the authors could find. However, Brazil was among the countries with the highest death tolls during early pandemic. Six of the 14 countries had decreased running activity counts in the period after 11 March compared with before 11 March, while the other eight countries had increased counts. The countries with the greatest increase were Switzerland, Netherlands, and United Kingdom, each having more than a 5% increase from the period before to the period after 11 March. Switzerland did not have any restrictions on gatherings or orders to shut down non-essential facilities, such as gyms. Netherlands and United Kingdom on the other hand had non-essential places closed but allowed outdoor exercise alone (Table S3).

#### 3.1.2. Time-Series Activity Frequency by Country

**Figure 4:**
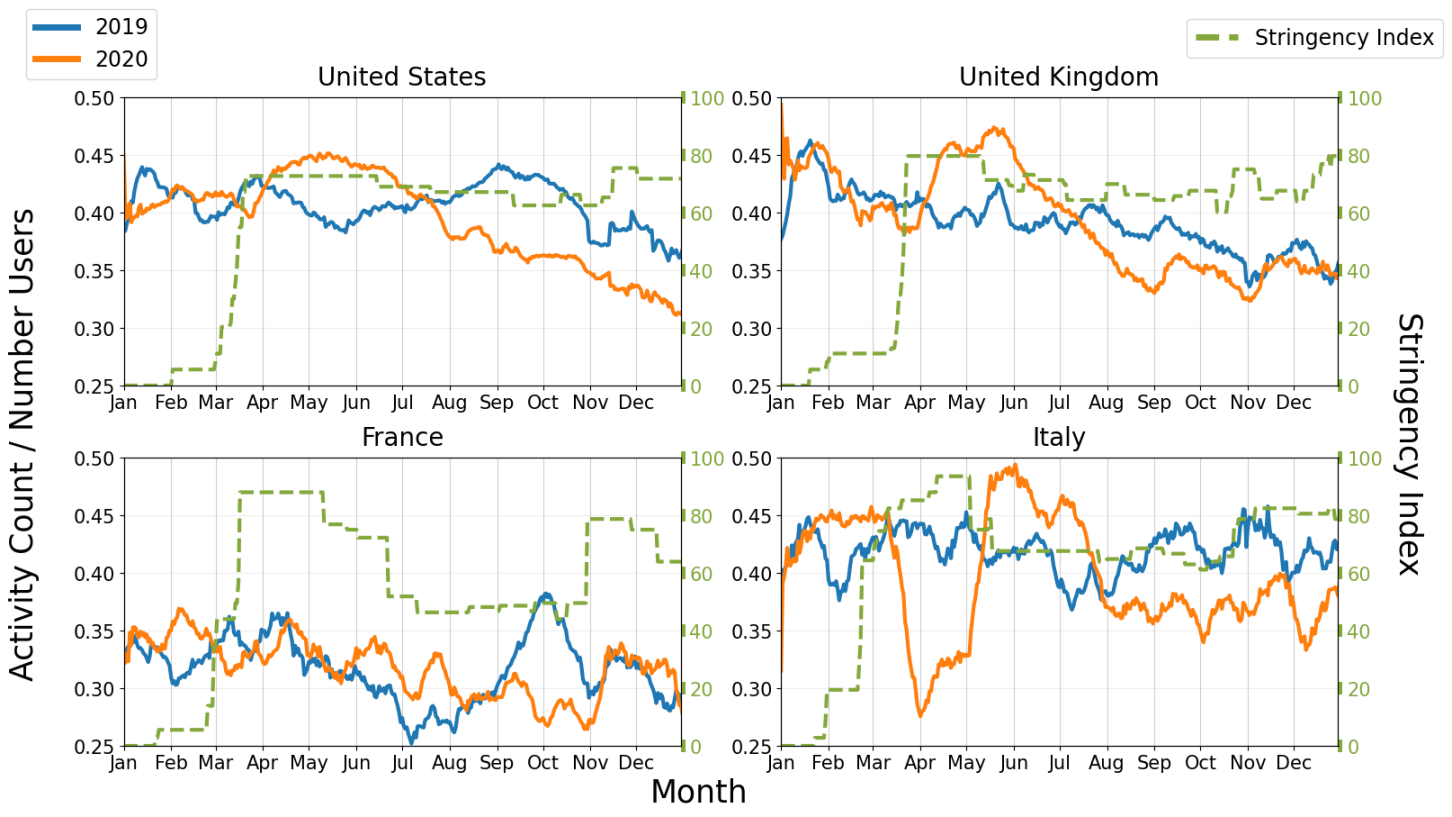
COVID-19 Government Response Stringency Index (GRSI) and daily activities counts per number of users in country using the controlled dataset.

Figure 4 display plots of activity counts and GRSI for the United States, United Kingdom, France, and Italy. Plots for all 14 countries that we analyzed are included in the supplementary materials (Figure S1). Figure 4 displays activity counts normalized by number of users for a given country, so activity levels can be directly compared between the pre-pandemic and the pandemic year and also between countries to identify which countries have residents who logged more or fewer activities than other countries’ residents.

At the onset of the first lockdown periods beginning mid-March of 2020, the United Kingdom and United States’ activity counts both decreased to below 2019 activity levels (Figure 4), immediately followed by an increase to activity levels which, for three months, were substantially higher than the corresponding 2019 activity counts. From September through the remainder of 2020, the slope of activity counts over time closely matched that of 2019, with activity counts trending down. Despite activity levels being higher in 2020 for three months following lockdown, they were generally substantially lower than 2019 levels for September through August for both countries.

France instituted a severe lockdown from 17 March to 11 May and again from 28 October to 15 December, where exercise was limited to within 1km of one’s residence for less than one hour per day during the first lockdown [26]. Interestingly, the activity levels of 2020 resemble that of 2019 for the period between March through June, suggesting that French users took advantage of their allotted 1-hour time to exercise during lockdowns, and lockdown did not have a large impact on the frequency of PA. In 2019, there was a large increase in activity counts in mid-September through mid-October which may be associated with seasonal change.

On 9 March, a national lockdown was issued in Italy, and on 22 March, all outdoor activities in public places such as parks and indoor facilities were banned. The GRSI indicates that restrictions in Italy were slightly more strict than in France. In late April, restrictions were eased and physical activity was allowed within 200 m of one’s residence [27]. We observe that running activity rates in Italy were stable for the beginning of March, then dropped from about 0.45 to 0.28 activities per user per day (Figure 4), corresponding with the lockdown and increased GRSI. After Italy allowed greater freedom of movement as represented by decreased GRSI on 4 May rates rose from 0.33 to 0.49 activities per user per day, reaching above pre-lockdown levels. Italy’s highest activity counts of 2020 occurred in May, right after lockdown was lifted.

Italy, France, and Spain reached the highest GRSI, respectively, among the 14 countries we examined (Figure S1). The highest GRSI for these countries occurred during the March-April lockdown periods, where GRSI fluctuated but remained close to 90 for each country. Figure 4 shows that during the March-April lockdowns, Italy’s activity reached a minimum for the year at 0.28 activities/user daily, while France’s activity levels remained mostly stable and closely matched 2019 activity levels with a minimum of 0.31 activities/user daily. Spain reached the lowest activity levels of all 14 countries in 2020 with 0.14 activities/user daily, despite having GRSI lower than France and Italy for most of March and April. We believe that policy differences play a large role. France let people exercise outdoors close to their home for limited time daily, while Spain and Italy both did not allow exercise in public places during their most restrictive lockdown periods.

### 3.2. COVID-19 and Timing of Exercise

COVID-19 pandemic policies caused widespread workplace and school closures, cancellation of events, and travel restrictions.

**Figure 5:**
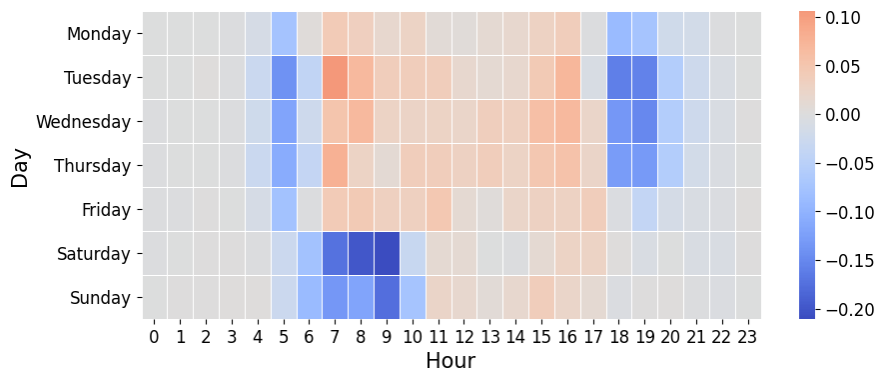
Positive (red) and negative (blue) absolute change in normalized activity counts from 2019 to 2020.

We examined distribution of time when users began their recorded activities following the WHO pandemic date of 11 March 2020 throughout the end of the year and correspondingly the same period in 2019. The day-hour time bins with the greatest difference in activity counts between the two years are shown in Figure 5. The time bins with the greatest decrease are between 7–10 AM on Saturday and Sunday and between 6–8 PM (hours 18–20 in Figure 5) on Tuesday, Wednesday, and Thursday. Time bins with the greatest increase are 7–9 AM on Tuesday and Wednesday, 7–8 AM on Thursday, and 4–5 PM on Tuesday, Wednesday, and Thursday. Time bins with a reduction in activity counts mostly fall outside of traditional weekday working hours, while time bins with increased counts are the hours within working hours and especially hours tightly hugging the beginning and end of working hours.

**Figure 6:**
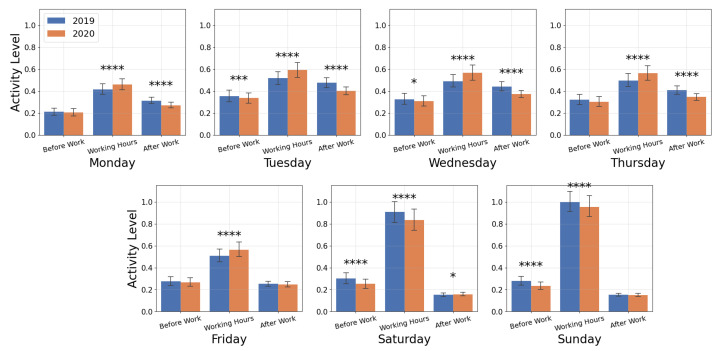
Normalized activity counts for the time periods before work (12 AM to 8 AM), during working hours (8 AM to 5 PM), and after work (5 PM to 12 AM). Significance with *p* < 0.05 is denoted by *, *** denotes *p* < 0.001, **** denotes *p* < 0.0001.

Figure 6 shows that in the pandemic year, all five weekdays had significantly more runs logged during normal working hours (*p* < 0.001), while weekends had significantly fewer runs during working hours, compared with the pre-pandemic year (*p* < 0.001). Only Tuesday, Saturday, and Sunday showed a significant decrease in runs logged before working-hours for 2020 compared with 2019 (*p* < 0.001). Monday, Tuesday, Wednesday, and Thursday also had significant decreases in activity counts after working hours for 2020 (*p* < 0.001).

We also examined the time of activity for each of the 14 countries. See Supplementary Materials for time-binned activity counts broken down by country and significance findings (Figure S2). The significant changes in time-binned activity shown in Figure 6 are generally reflected at the country level. Additional significant changes were detected for individual countries, for example Italy, Germany, and United Kingdom experienced increased activity before working hours on Monday (*p* < 0.05, *p* < 0.05, and *p* < 0.01, respectively). Other countries had significant changes that were opposite of the significant global trend identified in Figure 6. Notably, both France and Japan had a significant decrease in activities (*p* < 0.05 and *p* < 0.01, respectively) logged during working hours on Monday, and Spain experienced a decrease during working hours on Tuesday and Wednesday (*p* < 0.01 and *p* < 0.05, respectively) and a decrease after working hours on Saturday (*p* < 0.05).

## 4. Discussion

We used objective wearable device data for a retrospective analysis into physical activity behaviors of Stryd users (dedicated runners) prior to and during the COVID-19 pandemic. Currently, the body of research on PA during COVID-19 relies heavily on data collected via surveying people about their past PA habits and via objective device-tracking of step counts. Both can be very misleading, with survey data subject to recall bias and exaggeration, and step counts subject to user nonwear for dedicated athletic activities, such as playing soccer, climbing, or running where a user does not carry their mobile device [5,6,9,10]. Our analysis provides insight into a unique group of active individuals who purchased a footpod for the purpose of tracking more detailed information on their runs and overall running fitness. As such, we expect that running is a high priority activity for users, and they are also likely to be diligent about wearing the footpod for runs. Furthermore, as a retrospective analysis, we eliminate sources of behavior bias that can result when subjects are aware of their behavior being studied.

Overall, our analysis revealed variations across countries in PA behaviors of more competitive runners. It is possible that the pandemic influenced this group in a different way than others and we cannot extrapolate these behaviors to other groups. Countries enacted different pandemic policies, with Italy, France, and Spain respectively enacting the strictest lockdowns out of the 14 countries examined. Interestingly, Italy and Spain had the largest decreases in activity during lockdown periods while France had a relatively minimal decrease in activity counts. Previous work found that countries with higher government pandemic stringency were more likely to be insufficiently active [18]. France’s PA is notable and should be investigated further because France reached the second highest stringency index during their strict strict lockdown out of the countries we analyzed, but PA resembled the PA level of the pre-pandemic year. As discussed previously, France allowed outdoor exercise for residents close to their homes, while Spain and Italy did not allow public outdoor exercise.

Many countries experienced decreased PA during lockdown periods indicated by the spike in GRSI. Tison et al. examined smartphone step-counts during initial pandemic lockdowns and showed that Brazil, France, Italy, Japan, United Kingdom, and United States all experienced a decrease in steps comparing a pre-pandemic baseline on 11 February 2020 to every day in the period between 1 April and 1 June 2020 ranging from a 10% decrease to about a 47% decrease [12]. While step-counts were down, likely due to adherence to lockdown measures, shifting to remote work, and public closures, our work suggests that step-counts my not accurately reflective overall PA. Our work suggests that dedicated runners in Japan, United Kingdom, and United States increased logged PA from before to during the lockdown. These three countries had region-dependent restrictions, but generally gatherings were restricted and Japan and United Kingdom both nationally only allowed solo outdoor exercise (Table S3). Similarly, Venter et al. found an increase in recreational PA counts during initial lockdown using Strava data from 270,000 users logging runs, walks, hikes, and bike rides in Oslo, Norway [28]. More studies need to utilize logged activity data to assess its use and investigate the differences from step-counts and questionnaire data for measuring PA.

Previous work suggests that there was an increase in people working from home during the pandemic and increased sedentary behavior [5,29]. In our analysis of exercise timing throughout the week, we found that during 2020, users logged significantly more activities within standard working hours on Monday through Friday weekdays compared with 2019, and significantly fewer activities on weekends during the working hour period. Venter et al. similarly found that more running, hiking, and walking activities were logged during daylight hours, and fewer were logged in the mornings and evenings corresponding to this definitions of before work, working hours, and after work [28]. These effects may be due to the increased flexibility that came from pursuing work or education from home and the elimination of commuting. This may indicate that these times are preferred over times outside of working hours such as early morning or late evening, and it could also indicate that the reduced structure and increased flexibility of working at home may have contributed to decreased activity, or that people attempted to spread their exercise out over the day to avoid people and risk contracting the virus. Due to the large variation in pandemic workplace, school, public gathering, and other pandemic policy, COVID-19 case rates, health systems, and cultural and socioeconomic factors, it is not within the scope of this work to relate specific policies and cultural factors to observed PA behaviors within countries.

It is of widespread interest to determine whether working-from-home is a workplace policy that would promote healthy PA. Future work could investigate those who exercised outside of working hours before the pandemic and shifted to exercising during working hours during the pandemic and whether they experienced increased or decreased PA. Furthermore, future analysis could investigate whether activity increased from the pre-pandemic year of 2019 to later years of the pandemic, to reduce the affect of the initial shock and panic of the pandemic. Previous questionnaire-based research indicated that habitually active individuals experienced a decrease in PA, while less active individuals were more likely to have increased PA [5,17,23,24]. We are interested in whether this finding is supported by device-measured data, and if there is variation between countries.

This present study’s strengths include that it examines global PA and also the individual PA of 14 countries using objective data for direct comparison across countries and before to during the pandemic. Also, the study uses a large sample size of over 7000 subjects, reports analysis of logged physical activities instead of solely step-counts which most previous objective-tracking PA studies are limited by, and we cross-examine government pandemic response with PA levels. Limitations of this analysis are that it only explores the PA of dedicated runners and it cannot be extrapolated to other groups. Also, it cannot explain the changes in PA. Possible factors for decreased activity counts in 2020 are that the pandemic was an extremely stressful time due to economic instability, isolation, disruption of regular life, fear of contracting the virus, and government pandemic policy. Another explanation for lower activity logging is that there were widespread in-person race cancellations beginning in February, so users may have reduced their training due to race cancellations. The device strictly captures running activity of dedicated runners and misses other daily activity. Finally, our dataset may not have an accurate representation of socioeconomic groups who are less likely to own wearable devices.

## 5. Conclusions

This work aims to utilize wearable device data, an objective data source unavailable during previous pandemics, in order to investigate the physical activity of runners during the COVID-19 pandemic. Activity counts generally decreased from 2019 to 2020, although eight of 14 countries examined had increased counts from the period before to the period after the date that the WHO declared a pandemic. We found that during the pandemic more activities were logged during normal working hours than before the pandemic, and the highest decrease in activities occurred in certain times outside of normal working hours. Studying the exercise trends during the COVID-19 pandemic will enable policymakers and public health officials to consider how to implement policies which encourage exercise to contribute to the overall health of the population. Further identification of factors leading to the increases in PA identified in this work could also guide public health officials in promoting healthy exercising.

## Data Availability

Restrictions apply to the availability of these data. Data was obtained from Stryd and are available from the authors with the permission of Stryd.

## Supplementary Materials

The following supporting information can be downloaded at: https://www.mdpi.com/article/10.3390/ijerph191912933/s1, Table S1: Sample set of activity data recorded by the first author; Table S2: Percent increase in activity counts by country from 2019 to 2020 compared for 67-day periods before and after the WHO pandemic date of March 11.; Table S3: Description of the specific policies enacted during the most restrictive time periods to combat the first wave of the COVID-19 pandemic in 2020.; Figure S1: COVID-19 Government Response Stringency Index (GRSI) and daily activity counts per number of users for 14 countries in 2019 and 2020; Figure S2: Normalized activity counts for the time periods broken down by country.

## Abbreviations

The following abbreviations are used in this manuscript:

COVID-19	Coronavirus disease-2019
PA	physical activity
GPS	Global Positioning System
UTC	Coordinated Universal Time
GRSI	Government Response Stringency Index
WHO	World Health Organization
AC	activity counts
