# Supplementary material for: Global Impact of COVID-19 Pandemic on Physical Activity Habits of Competitive Runners: An Analysis of Wearable Device Data
Source: Int J Environ Res Public Health. 2022 Oct 10;19(19):12933. doi: 10.3390/ijerph191912933 (PMC9566411; doi:10.3390/ijerph191912933)
Supplement: Supplementary file 1 [file ijerph-19-12933-s001.zip › ijerph-1920468-supplementary.pdf]

Supplementary File 1

Table S1. Sample set of activity data recorded by the first author. A select number of attributes are shown in this table due to space limitations. Each individual activity is represented by a single row in the table.

user_id	name	ftp (W)	timestamp	Distance (m)	moving_time (sec)	elapsed_time (sec)	average_speed (m/s)	stress	average_power (W)	average_cadence (spm)	average_heart_rate (bpm)
X	Morning Run	185.2708591	1608044924	1781.51001	613	1650.327026	2.856778145	10.3141778	138.1538462	178	148
X	Threshold Workout	185.2708591	1608143595	15489.08008	4309	4571.477051	3.564163788	167.7299361	194.6999767	187	97
X	Afternoon Run	201.1195053	1608245064	9838.669648	3050	3094.309082	3.215332901	64.36757416	180.8224115	184	162
X	7 x 1mi @ over marathon pace	201.1195053	1608329749	15186.11035	4286	4549.436035	3.544572634	122.9287391	192.1865394	187	163
X	Afternoon Run	201.1195053	1608409095	9844.129883	4979	5258.838867	1.911313366	53.09201935	138.5385233	151	146
X	Afternoon Run	202.8856033	1608680371	5241.939941	1725	1901.501953	3.044168309	35.44876141	179.4450231	177	152
X	Morning Run	202.8856033	1608748700	8744.759922	2602	2718.52002	3.375760649	82.55586384	204.2854399	183	172

Table S2. Percent increase in activity counts by country from 2019 to 2020 compared for 67-day periods before and after the WHO pandemic date of March 11. Values correspond to **Figure 2**.

Country	% Increase Pre-March 11	% Increase Post-March 11	Difference
Spain	1.44	-42.39	-43.83
Italy	5.68	-19.37	-25.05
Brazil	-0.77	-15.18	-14.41
France	2.68	-2.30	-4.98
Belgium	11.15	8.37	-2.78
Austria	22.31	20.869	-1.41
Canada	-2.42	-1.47	0.95
Australia	-2.17	-0.30	1.87
Japan	-2.67	-0.50	2.17
Germany	2.80	6.84	4.05
United States	-2.07	3.72	5.79
United Kingdom	-2.16	5.35	7.51
Netherlands	3.81	17.05	13.24
Switzerland	-4.06	18.22	22.28

Table S3. Description of the specific policies enacted during the most restrictive time periods to combat the first wave of the COVID-19 pandemic in 2020. Policy information was taken from the Oxford COVID-19 Government Response Tracker.

Country	Level	Period	Description of Policies
**Australia**	Regional+ National	Apr 1 - Apr 14	• School closures by region<br>• National non-essential places closed and public events cancelled<br>• Stay at home and WFH except essential travel<br>• No non-essential gatherings over 2 people
**Austria**	Highly National	Mar 16 - Apr 13	• National school closures for those 15 and older<br>• Non-essential stores and workplaces closed; WFH encouraged; events banned<br>• Leaving home banned except for activity and essential activities<br>• No gatherings over 5 people
**Belgium**	Highly National	Mar 2 - May 4	• School closures<br>• Companies must shift to WFH for every possible position<br>• Stay at home order except essential movement (includes work). No outdoor exercise in public places.<br>• No gatherings over 2 people
**Brazil**	Highly Regional	May 5 - May 31	• All schools closed due to local/regional policies<br>• Non-essential businesses closed due to local/regional policy<br>• Ministry of Health says local gov will adopt their own strategy for pandemic, so complete lack of federal policy<br>• No restrictions on outdoor exercise
**Canada**	Highly Regional	Apr 1 - Apr 3	• All public schools closed<br>• Most territories required non-essential business closures<br>• National ban on gatherings 50+ people, more restrictive local measures<br>• Certain territories requiring stay at home but allow exercise outdoors
**France**	Highly National	Mar 17 - Apr 25	• All schools closed<br>• Non-essential business closures, ban on public and private events<br>• No gatherings more than 5 people<br>• Recommend all residents to stay home. Enacted a supervision system with people allowed to exercise outdoors within 1 km from home and less than 1 hour.
**Germany**	Regional+ National	Mar 23 - May 2	• Schools closed by local/regional policy. Regional stay at home orders.<br>• Close non-essential shops and facilities; social distancing required in other business/workplaces<br>• Close leisure parks and activities, indoor and outdoor. Outdoor exercise with 1 other person is fine outdoors<br>• Ban on public events, gatherings over 2 people

Country	Level	Period	Description of Policies
**Italy**	Highly National	Mar 30 - May 3	• One of Europe's tightest lockdowns<br>• All schools/universities closed. Non-essential shops, leisure, and sports areas, workplaces closed; WFH when possible<br>• Mandate stay at home except work, medical reasons, and food. Not allowed to meet anyone.<br>• No outdoor exercise allowed; children not allowed outside at all
**Japan**	Regional+ National	Mar 17 - May 10	• School closures. Non-essential commercial activity closed including fitness centers<br>• Ban on gatherings over 100 people; more local restrictions. Seeing others is forbidden except work, groceries, medical<br>• Solo outdoor exercise allowed<br>• Need a form to leave the house
**Netherlands**	Highly National	Mar 24 - May 10	• School closures. Non-essential commercial activity closed including fitness centers<br>• Ban on gatherings over 100 people; more local restrictions. Seeing others is forbidden except work, groceries, medical<br>• Solo outdoor exercise allowed<br>• Need a form to leave the house
**Spain**	Highly National	Apr 12 - May 3	• Schools closed nationwide<br>• National lockdown; must stay at home and seek permission for essential travel<br>• Ban on public even<br>• People were not allowed on public streets for exercise
**Switzerland**	Regional+ National	Apr 16 - May 13	• No strict enforcements on gathering. Government asks public to stay at home and not gather.<br>• Gov asks to WFH, social distance in workplaces<br>• State of emergency order
**United Kingdom**	Regional+ National	Mar 23 - May 10	• All schools close<br>• Public-facing businesses, fitness clubs, social venues close. WFH recommended.<br>• Public transit reduced service capacity<br>• National stay at home order except work, exercise alone, essential reasons
**United States**	Highly Regional	Mar 21 - Jun 14	• All states order schools to close with exceptions<br>• States implement stay at home policies; shut down non-essential workplaces<br>• State bans on gatherings

Figure S1. COVID-19 Government Response Stringency Index (GRSI) and daily activity counts per number of users for 14 countries in 2019 and 2020.

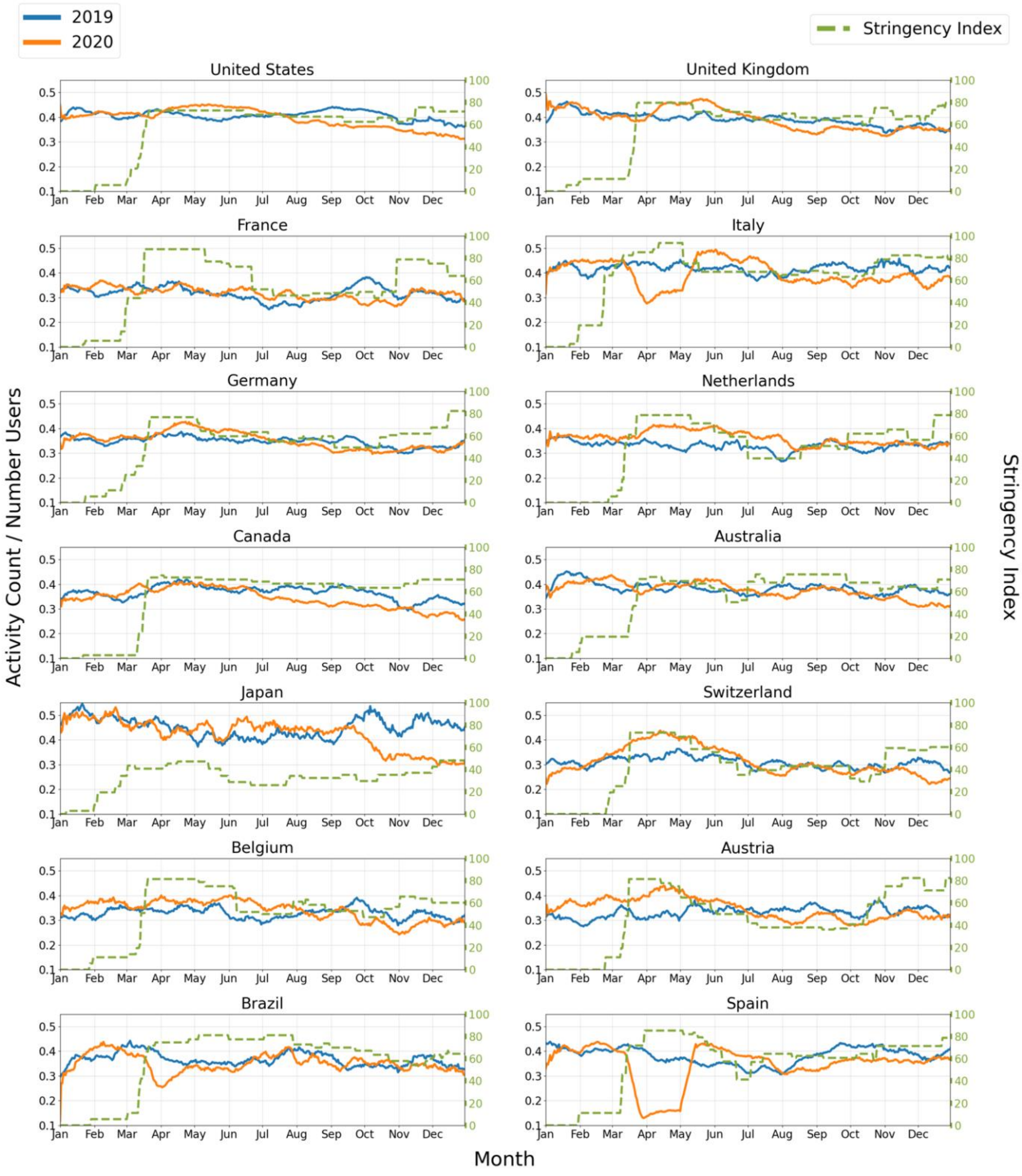

Figure S2. Normalized activity counts for the time periods before work (12 AM to 8 AM), during working hours (8 AM to 5 PM), and after work (5 PM to 12 AM). Significance with p<0.05 is denoted by *, ** denotes p<, *** denotes p<0.001, **** denotes p<.0001 for 14 countries in 2019 and 2020.

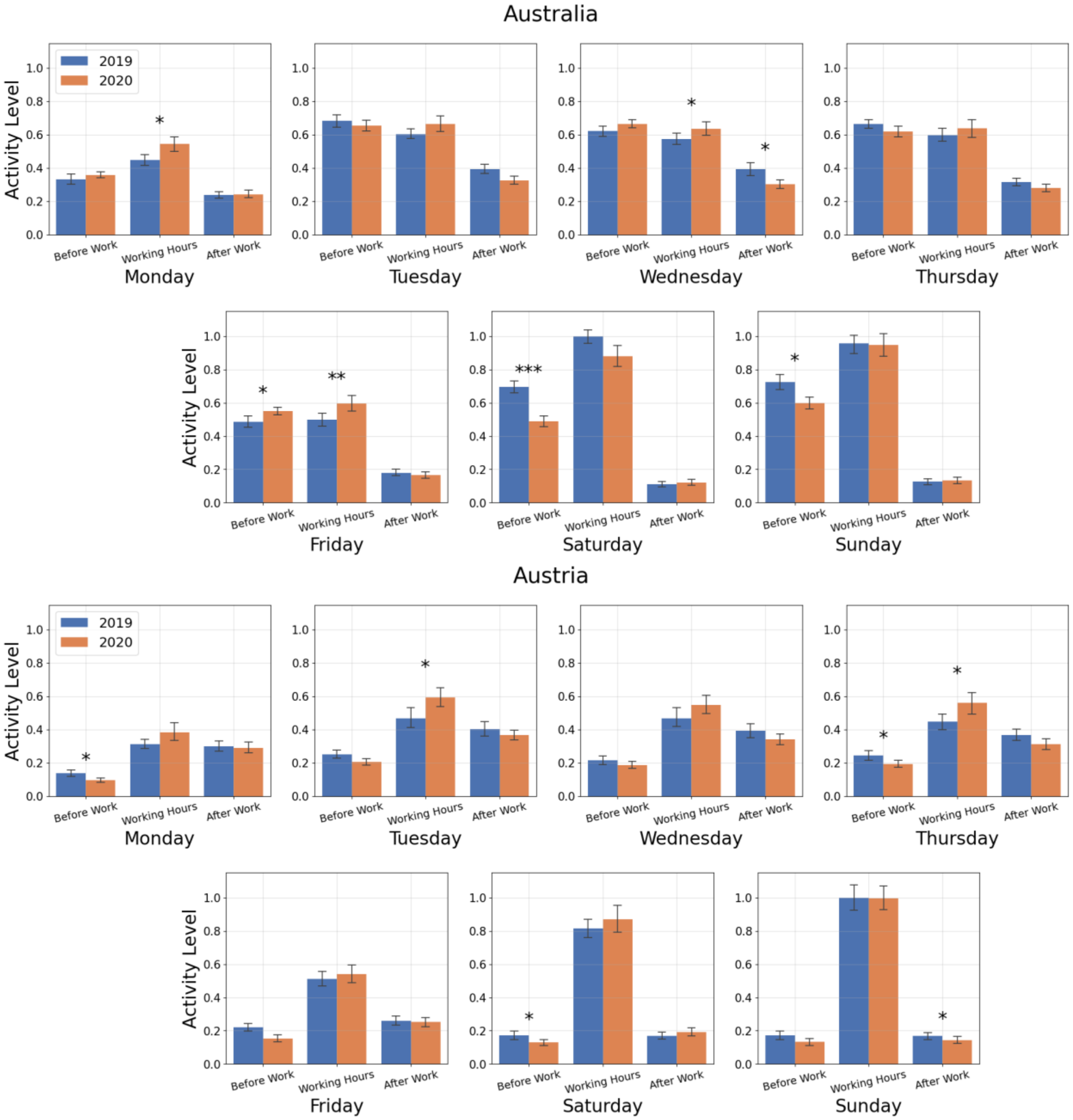

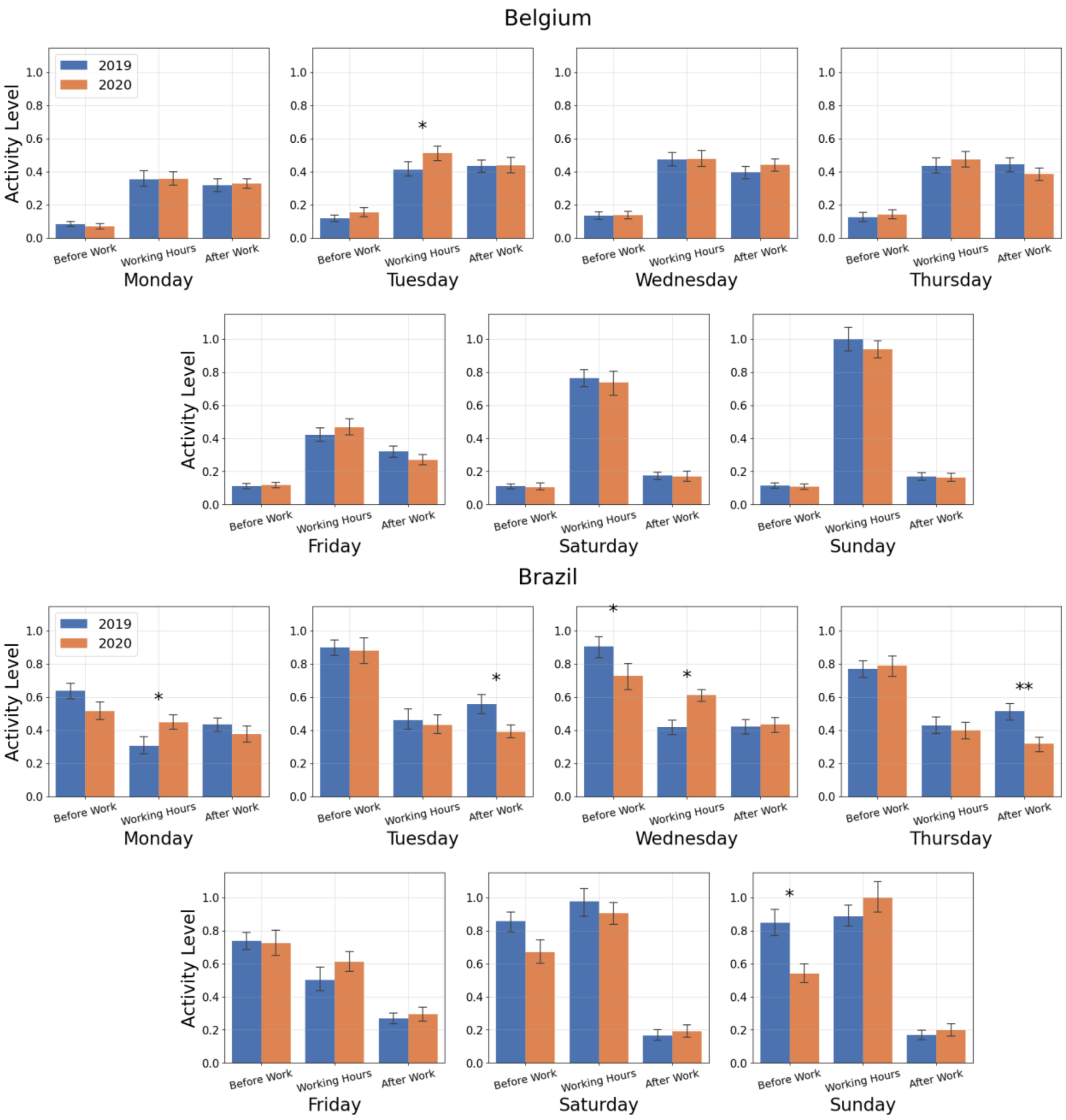
Belgium
Brazil
2019
2020
Activity Level
1.0
0.8
0.6
0.4
0.2
0.0
Before Work
Working Hours
After Work
Monday
Tuesday
Wednesday
Thursday
Friday
Saturday
Sunday
*
**

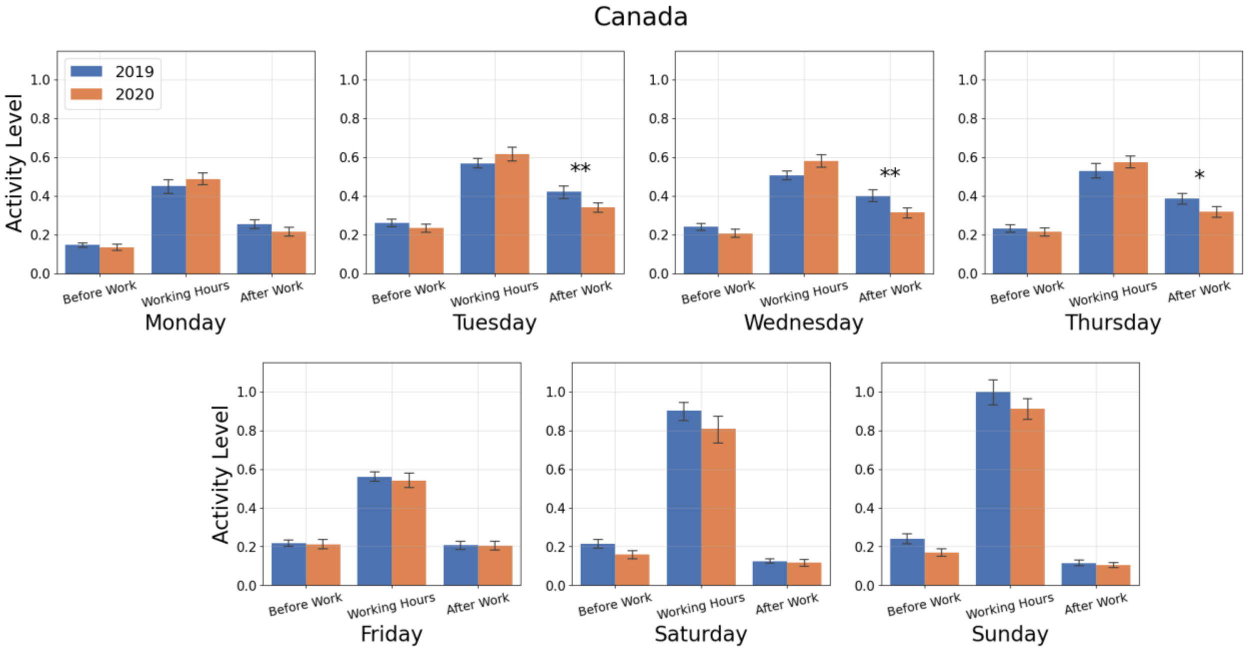
Canada
2019
2020
Activity Level
Before Work
Working Hours
After Work
Monday
Tuesday
Wednesday
Thursday
Friday
Saturday
Sunday
**
**
*

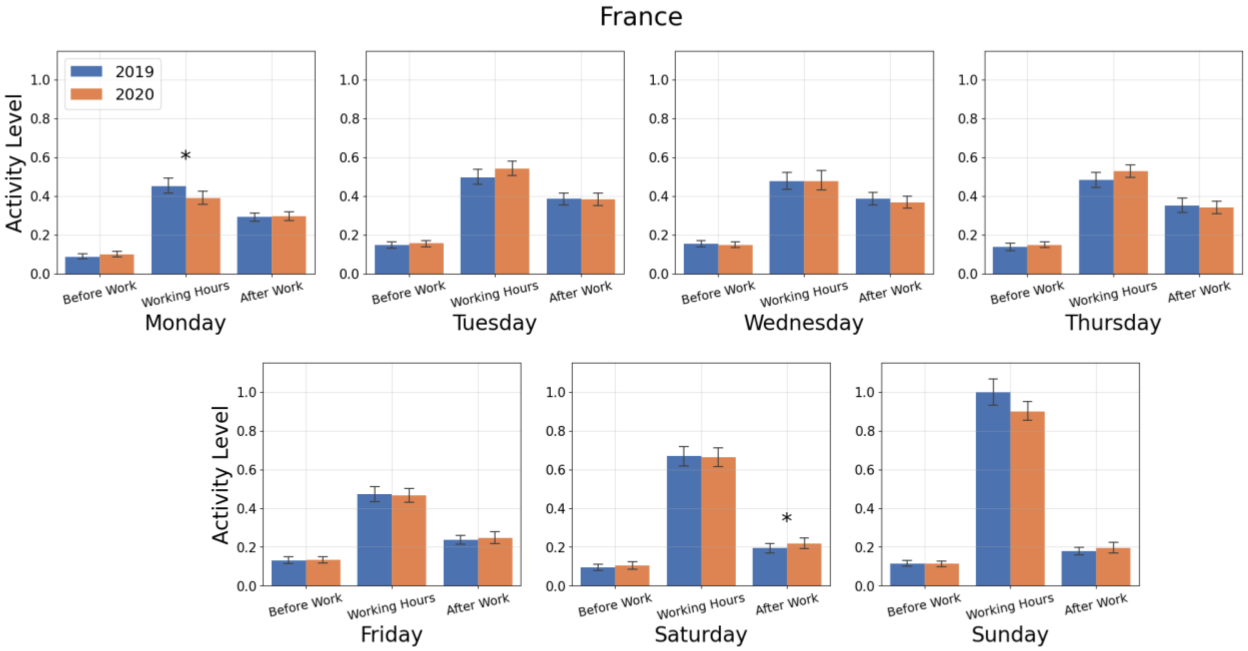
France
2019
2020
Activity Level
Before Work
Working Hours
After Work
Monday
Tuesday
Wednesday
Thursday
Friday
Saturday
Sunday
*
*

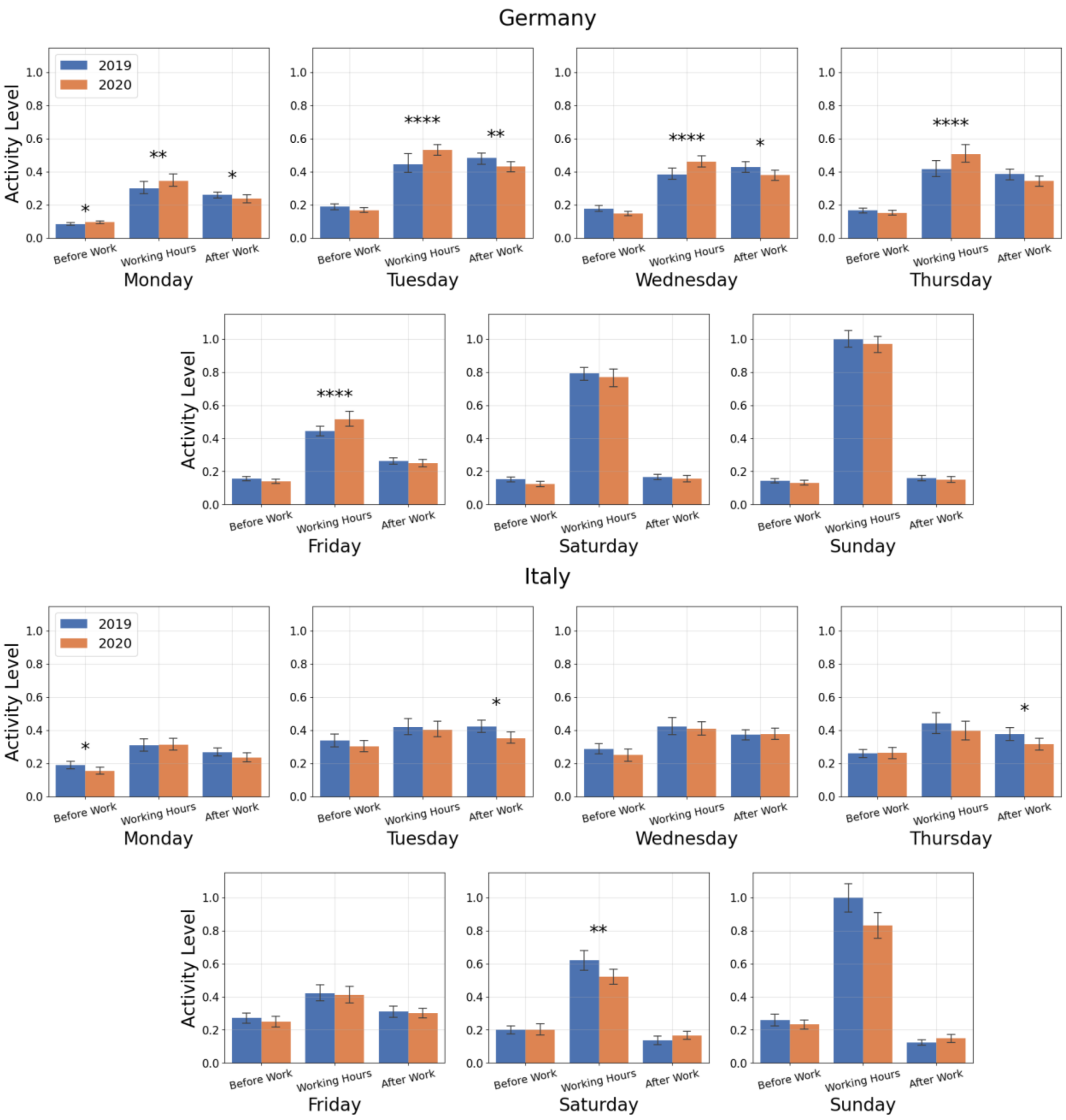

Germany
Italy
2019
2020
Activity Level
0.0
0.2
0.4
0.6
0.8
1.0
Before Work
Working Hours
After Work
Monday
Tuesday
Wednesday
Thursday
Friday
Saturday
Sunday
*
**
****

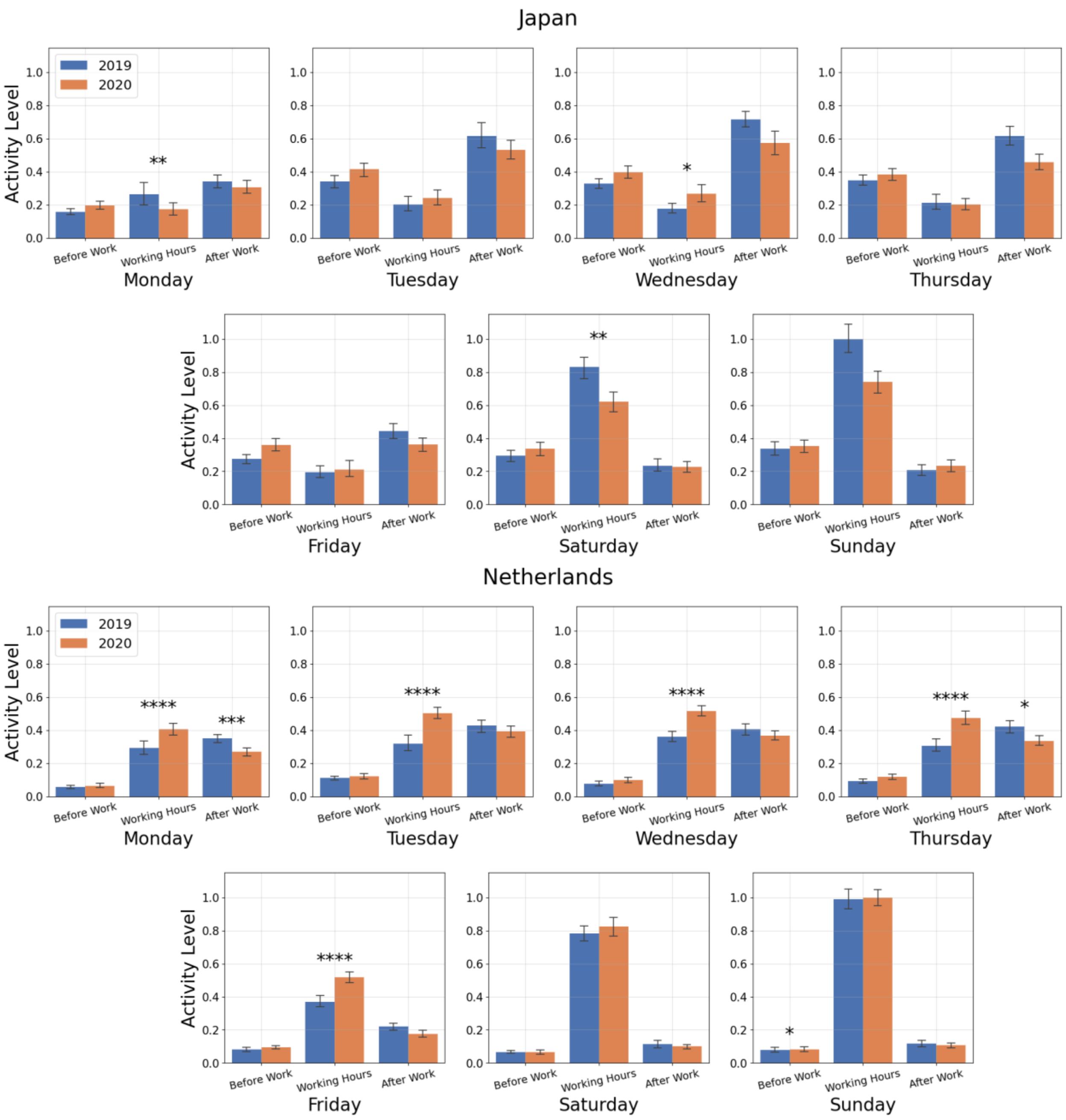
Japan
2019
2020
Activity Level
1.0
0.8
0.6
0.4
0.2
0.0
Before Work
Working Hours
After Work
Monday
Tuesday
Wednesday
Thursday
Friday
Saturday
Sunday
Netherlands

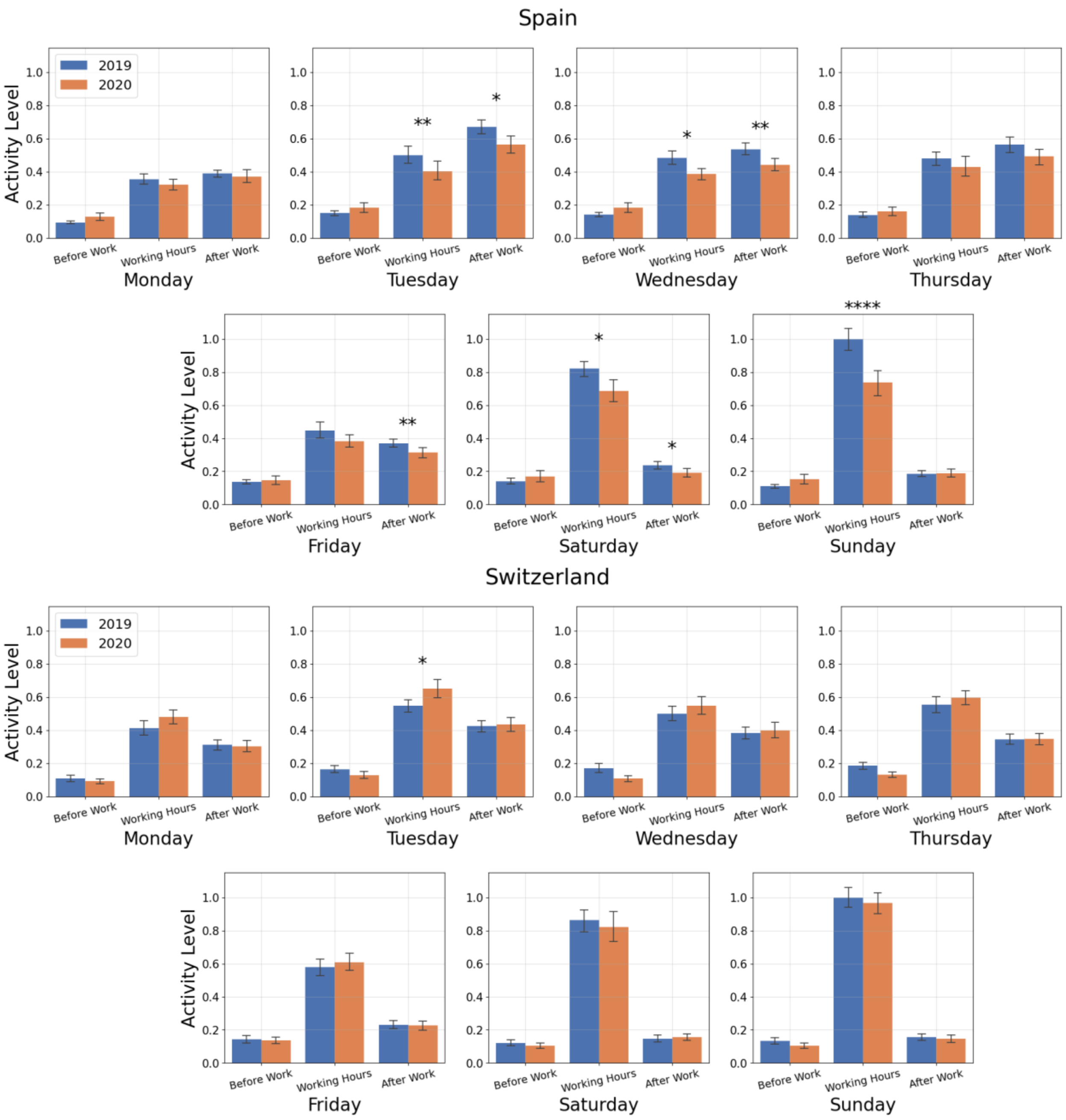
Spain
Switzerland
2019
2020
Activity Level
Before Work
Working Hours
After Work
Monday
Tuesday
Wednesday
Thursday
Friday
Saturday
Sunday
*
**
****
0.0
0.2
0.4
0.6
0.8
1.0

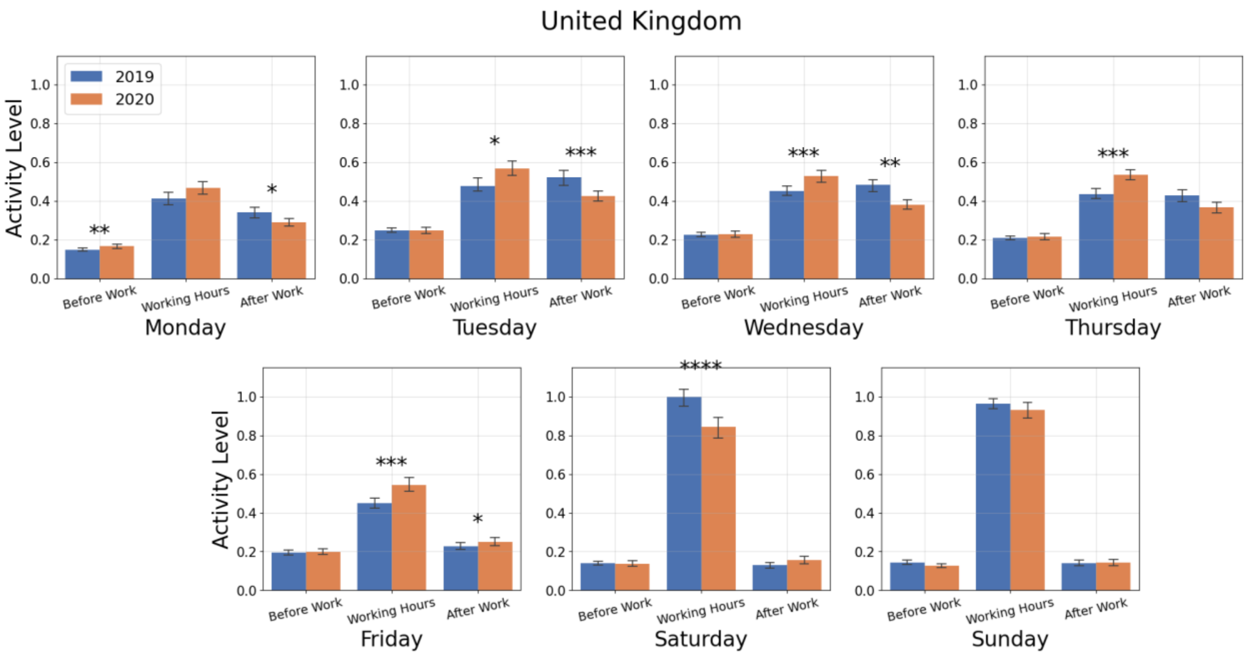
United Kingdom
2019
2020
Activity Level
Before Work
Working Hours
After Work
Monday
Tuesday
Wednesday
Thursday
Friday
Saturday
Sunday

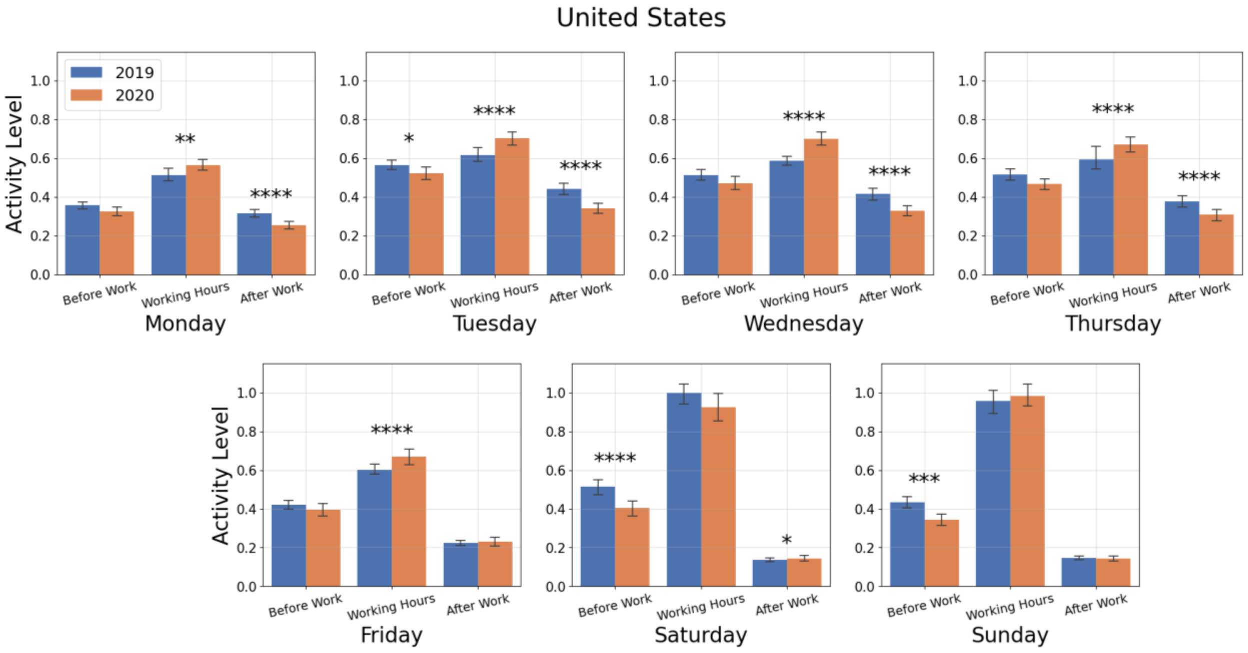
United States
2019
2020
Activity Level
Before Work
Working Hours
After Work
Monday
Tuesday
Wednesday
Thursday
Friday
Saturday
Sunday
